# Numerical Simulation and Structural Optimization of the Inclined Oil/Water Separator

**DOI:** 10.1371/journal.pone.0124095

**Published:** 2015-04-13

**Authors:** Liqiong Chen, Shijuan Wu, Hongfang Lu, Kun Huang, Lijie Zhao

**Affiliations:** 1 School of Petroleum Engineering, Southwest Petroleum University, Chengdu, Sichuan, China; 2 College of Geosciences, State Key Laboratory of Petroleum Resources and Prospecting, China University of Petroleum, Beijing, China; North China Electric Power University, CHINA

## Abstract

Improving the separation efficiency of the inclined oil/water separator, a new type of gravity separation equipment, is of great importance. In order to obtain a comprehensive understanding of the internal flow field of the separation process of oil and water within this separator, a numerical simulation based on Euler multiphase flow analysis and the realizable *k-ε* two equation turbulence model was executed using Fluent software. The optimal value ranges of the separator’s various structural parameters used in the numerical simulation were selected through orthogonal array experiments. A field experiment on the separator was conducted with optimized structural parameters in order to validate the reliability of the numerical simulation results. The research results indicated that the horizontal position of the dispenser, the hole number, and the diameter had significant effects on the oil/water separation efficiency, and that the longitudinal position of the dispenser and the position of the weir plate had insignificant effects on the oil/water separation efficiency. The optimal structural parameters obtained through the orthogonal array experiments resulted in an oil/water separation efficiency of up to 95%, which was 4.996% greater than that realized by the original structural parameters.

## Introduction

The changes in crude oil components and operating conditions resulting from oilfield development have created problems with oilfield production and crude oil gathering, transportation, and storage. The inability of conventional oil/water separators to adapt to today’s high water content is a major issue that could be effectively solved by converting horizontal separators into inclined separators and, thereby, improving separation efficiency.

In 2006, relevant studies were performed on the inclined free water separator in China’s Daqing Oilfield. Indoor simulations and field experiments were conducted, resulting in the development of a Φ 2.0 × 20 m inclined separator, which was applied in 2009 [1].

In 2008, the Southwest Petroleum University proposed a design concept for an inclined-plate, gas-liquid separator. Studies have shown that this separator has a simple structure, good separation effects, and a short separation time [2].

Researchers have conducted a plethora of studies on the inclined oil/water separator. However, due to the large number of factors that influence oil/water separation and the diversity of separator structures, a comprehensive set of design theories and methods has not been developed. The design of the inclined oil/water separator, according to empirical data alone, often cannot achieve the best separation effects. Therefore, in order to improve the efficiency of oil/water separation, comprehensive studies on the internal flow field of inclined oil/water separators and the motion of oil droplets are necessary [3–5].

In recent years, an increasing number of scholars have applied computational fluid dynamics (CFD) to studies on oil/water separation and have used CFD software to optimize the structure of oil/water separation equipment [6, 7]. He Limin from the China University of Petroleum used the volume of fluid (VOF) model to study the oil/water two-phase flow and obtained the critical particle size range for separation equipment [8, 9]. Hou Xianrui from Dalian Maritime University used the Mixture model to simulate the effects of the inlet component, the baffle, and the coalescing component on the separation performance of gravity oil/water separation equipment [10]. Zhang Li from Tianjin University used the Laminar model to simulate the effects of the inlet component and baffle on the separation performance of gravity oil/water separation equipment and optimized the structure of oil/water separation equipment based on the simulation results [11].

In this study, a mathematical model was created based on basic inclined separator theory. Fluent software was used to execute a numerical simulation of an inclined separator. Based on the simulation results, an orthogonal array of experiments was performed in order to optimize the structure of the inclined separator, and a field experiment was executed in order to validate the separation efficiency of the optimized separator.

## Basic Theory

### Separation Principle

**Fig 1:**
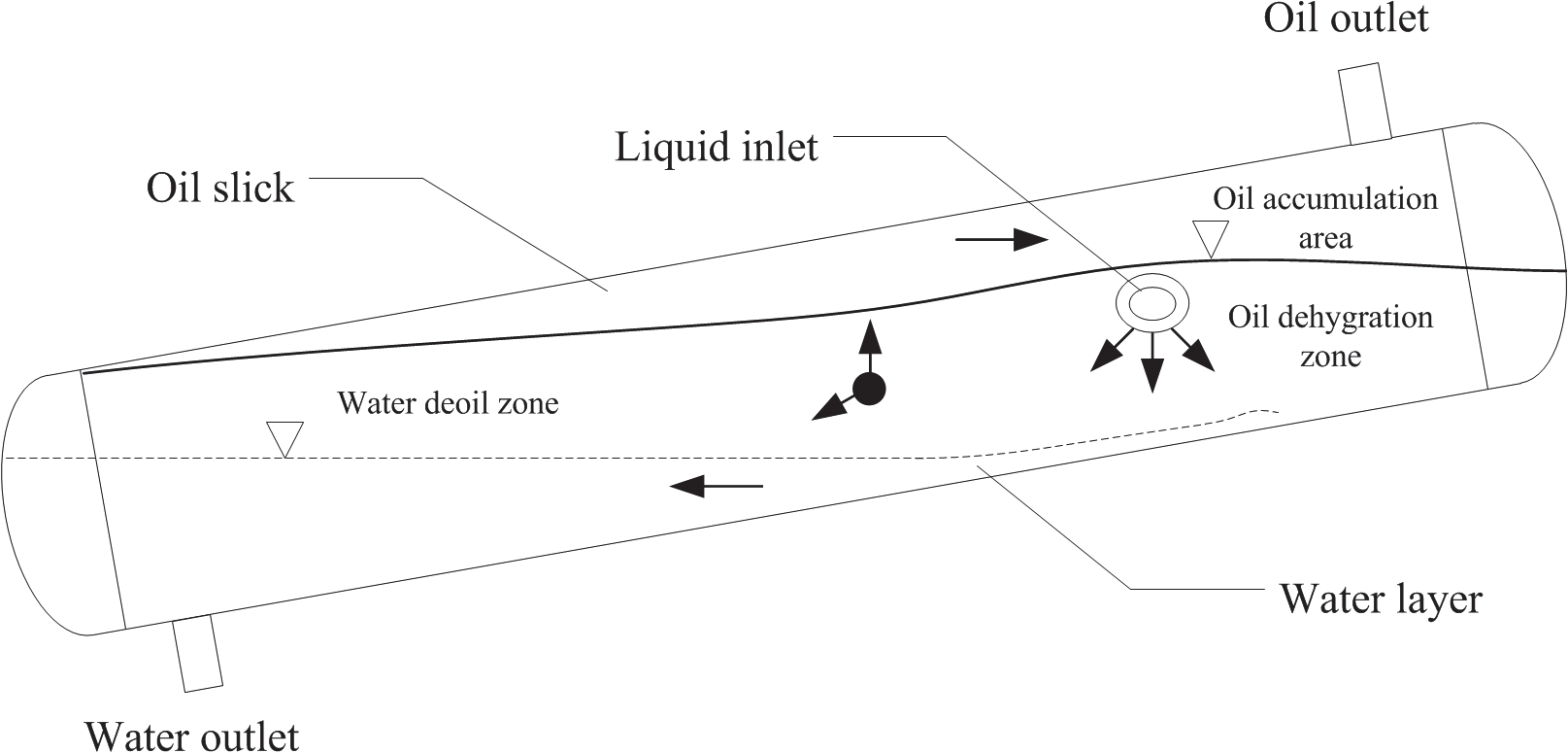
The separation principle of the inclined oil/water separator.

The inclined oil/water separator is an oil/water separation device that uses the buoyancy separation method; the separation principle of this separator is shown in **Fig 1**. After a large volume of liquid enters the device, the oil phase gathers in the upper portion of the separator, and the water phase gathers in the lower portion of the separator. Next, gravity separation occurs in the oil phase gathering section, and buoyancy separation occurs in the water phase gathering section. Because the water phase comprises the majority of the liquid, buoyancy separation is the primary separation process of the produced liquid [1].

### Main components

**Fig 2:**
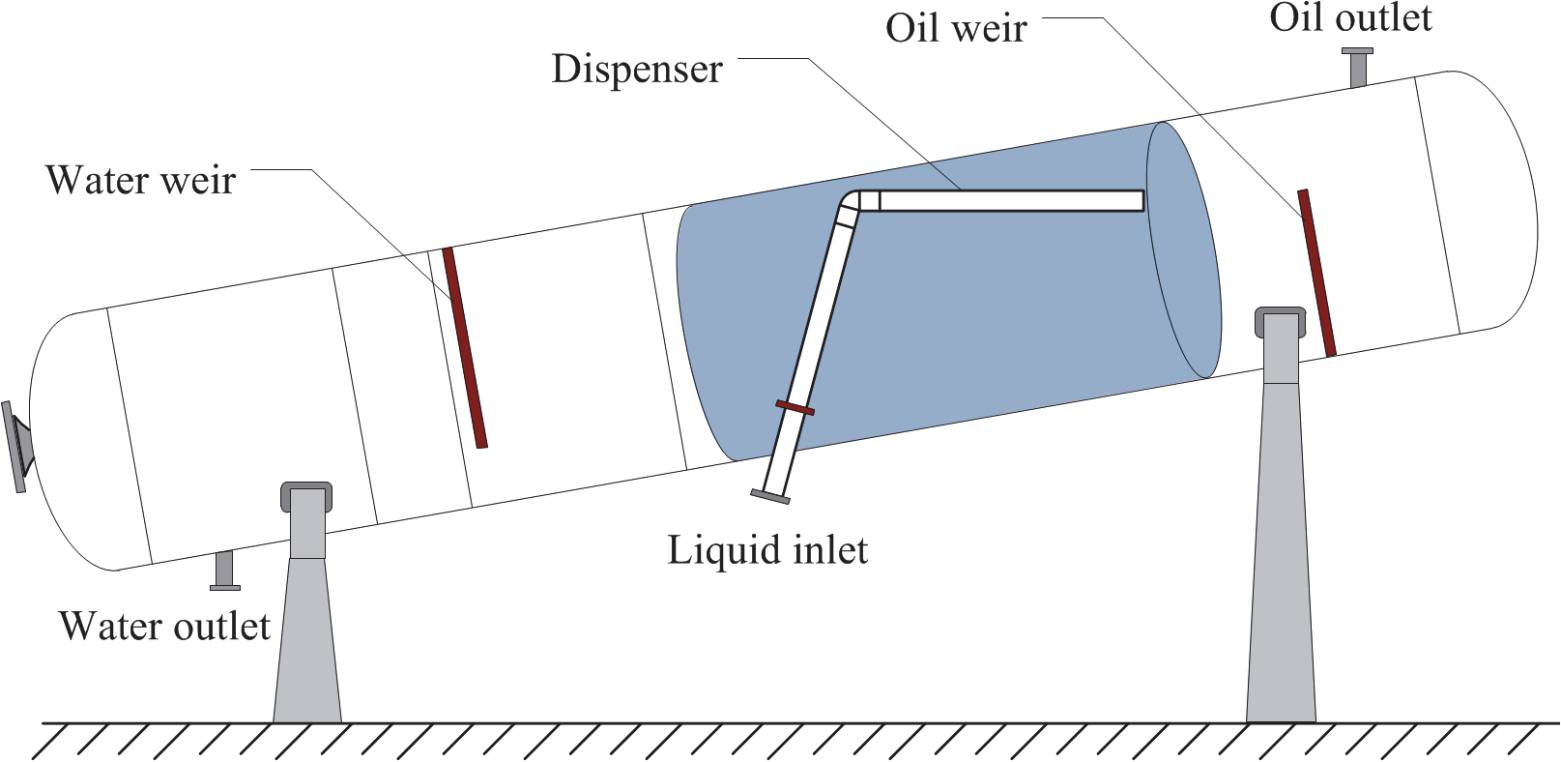
The schematic diagram of the inclined oil/water separator.

The inclined oil/water separator primarily consists of the separation tank, dispenser, oil outlet, water outlet, oil weir, and water weir; its schematic diagram is shown in **Fig 2**.

Currently, most separator dispensers are uniformly distributed round holes with equal diameters.

The weir plates are mostly used to regulate the oil/water interface within the separation tank. The horizontal positions of the oil and water weirs in the oil/water separator often range between 0.5 D and 1.5 m, where D is the diameter of the separator.

The oil outlet is generally located at the bottom, top, or wall of the separation tank. The water outlet is located at the left side of the bottom of the separation tank [1].

### Separation efficiency

The separation performance of an oil/water separator is often represented with the separation efficiency, *ε*, which is defined as

$$\varepsilon =1-\frac{C_{d}}{C_{i}}$$ 1

where *C*_*d*_ is the water outlet oil content;*C*_*i*_ is the inlet oil content.

## Mathematical Model

Based on Euler multiphase flow analysis, the realizable *k-ε* two equation turbulence models was used to perform a numerical simulation of the oil and water separation process of an inclined separator. This necessitated a mathematical model [12, 13].

The flow control equations for oil and water separation are the continuity and momentum equations. The universal control equation for turbulent incompressible fluids is

$$\frac{\partial (\rho \Phi )}{\partial t}+\nabla (\rho u\Phi )=\nabla (\Gamma \text{grad}(\Phi ))+S$$ 2

where *ρ* is the fluid density, kg/m^3^); *Φ* is a universal variable that can represent velocity and temperature; ***u*** is the velocity vector, m/s); *Γ* is the generalized diffusion coefficient; *S* is a generalized source term.

The relationships of the continuity, momentum, turbulent kinetic energy, and dissipation rate equations with common symbols are shown in **Table 1**.

**Table 1:** The concrete forms of the symbols in the universal control equations for turbulence.

Equation name	Common symbols		
	*Φ*	*Γ*	*S*
Continuity equation	1	0	0
Momentum equation	*u*_*i*_	*μ*_eff_ = *μ*+*μ*_*t*_	$-\frac{\partial p}{\partial i}+\frac{\partial }{\partial x}(\mu_{\text{eff}}\frac{\partial u_{x}}{\partial i})+\frac{\partial }{\partial y}(\mu_{\text{eff}}\frac{\partial u_{y}}{\partial i})+\frac{\partial }{\partial z}(\mu_{\text{eff}}\frac{\partial u_{z}}{\partial i})+S_{i}$
Turbulent kinetic energy equation	*k*	*μ+μ*_*t*_/*σ*_k_	*G*_*k*_+*G*_*b*_−*ρε*−*Y*_*M*_
Dissipation rate equation	*ε*	*μ+μ*_*t*_/*σ*_ε_	$\rho C_{1}E\varepsilon -\rho C_{2}\frac{\varepsilon^{2}}{k+\sqrt{\nu \varepsilon }}+C_{1\varepsilon }\frac{\varepsilon }{k}C_{3\varepsilon }G_{b}$

where *i* = *x*, *y*, *z*; *μ*_eff_ is the effective viscosity, Pa·s; *μ* is the dynamic viscosity, Pa·s; *μ*_t_ is the turbulent viscosity, Pa·s; *u*_*x*_, *u*_*y*_, *u*_*z*_ are the velocity components in the *x*, *y*, and *z* directions, m/s; *p* is the fluid pressure, Pa; *S*_*x*_, *S*_*y*_, *S*_*z*_ are the source terms, which have different expressions under different conditions; *k* is the turbulent kinetic energy per unit mass, m^2^/s^2^; *σ*_k_ is the Prandtl number for turbulent kinetic energy, dimensionless, whose value is 1.0; *G*_k_ is the production term for turbulent kinetic energy caused by the average velocity gradient, N/ (m^2^·s); *G*_b_ is the production term for turbulent kinetic energy due to buoyancy, N/ (m^2^·s); *ε* is the rate of dissipation of turbulent kinetic energy per unit mass, m^2^/s^3^; *Y*_*M*_ is the dilatation dissipation, kg/ (m·s^3^); *σ*_ε_ is the turbulent Prandtl number for dissipation rate, dimensionless, whose value is 1.2; *C*_1_ is a dimensionless constant; *E* is the time-average strain rate, s^-1^; *ν* is the coefficient of molecular dynamic viscosity, m^2^/s; *C*_1ε_, *C*_3ε_, *C*_2_ are empirical constants, dimensionless, with values of *C*_1ε_ = 1.44, *C*_3ε_ = 0.09, and *C*_2_ = 1.9.

## Case Study

### Basic data for the separator

The handling capacity of the inclined separator analyzed in this study was 300 m^3^/d, with a residence time of 6 min and an oil content of 10%. The diameter of the mixture inlet was 70 mm, the diameter of the water outlet was 50 mm, and the diameter of the oil outlet was 50 mm [13]. The concrete geometrical dimensions of the established model are shown in **Table 2**.

**Table 2:** The geometrical dimensions of the model.

Basic parameters	Dimension	Basic parameters	Dimension
Length of the separation tank (m)	7.78	Diameter of the liquid inlet (m)	0.07
Diameter of the separation tank (m)	0.44	Length of the oil outlet (m)	0.1
Volume of the separation tank (m^3^)	1.18	Diameter of the water outlet (m)	0.05
Length of the dispenser (m)	0.7	Length of the water outlet (m)	0.1
Diameter of the dispenser (m)	0.07	Height of the oil weir (m)	0.8D
Hole number of the dispenser	7	Position of the oil weir (m)	1.0D
Hole diameter of the dispenser	0.02	Height of the water weir (m)	0.5D
Hole spacing	3.0d	Position of the water weir (m)	1.0D
Longitudinal position of the dispenser	0.5D	Long radius of the head (m)	0.22
Horizontal (x) position of the dispenser	75%L_e_	Short radius of the head (m)	0.11

Note: D represents the diameter of the separator body; d represents the hole diameter of the dispenser; and L_e_ represents the length of the major separation region of the separator, namely the distance between the weirs.

### Inlet boundary conditions

The inlet was defined as the velocity inlet. The flow velocity at the inlet was 0.9 m/s (based on the actual conditions, the corresponding volume flow rate was 300.00 m^3^/d), and the velocity at the inlet was assumed to be uniformly distributed. The turbulent intensity at the inlet was 4.49%. The hydraulic diameter was 70.00 mm.

### Outlet boundary conditions

The free outflow boundary condition was applied at the outlet. Volume flow rate fraction at oil outlet is 10%, and volume flow rate fraction at water outlet is 90%.

### Wall boundary conditions

The no-slip boundary condition was used on the wall of the separator; thus, the wall was static. The fluid and turbulent velocities on the wall were equal to zero. The wall toughness was set as 0.15 mm.

### Meshing of the model

The structure of the inclined oil/water separator simulation model was large and complex, and adaptive meshing was adopted in Gambit. In order to ensure the precision and adaptability of the meshing, the mesh density was set to 2.5 mm × 2.5 mm. The entire computational domain consisted of 483,612 cells, and the dispenser mesh consisted of 5,149 cells.

### Physical parameters

The medium used for the purposes of this simulation consisted of a mixture of water and crude oil; the water content was 90%. The density and dynamic viscosity of water are 1000 kg/m^3^ and 1 mPa·s, respectively, and the density and dynamic viscosity of crude oil are 870 kg/m^3^ and 51 mPa·s, respectively.

## Numerical Simulation Results

### Separation efficiency

In order to study the separation performance of the inclined oil/water separator, numerical simulations were conducted at varying inclinations, ranging from 0° to 30°. Statistics regarding the water volume content at the oil outlet, the oil volume content at the water outlet, and the oil/water separation efficiency of the model at varying inclinations are shown in **S1 Table**.

According to **S1 Table**, when the inclination ranged from 0° to 12°, the oil/water separation efficiency increased as the inclination increased; when the inclination ranged from 15° to 25°, the oil/water separation efficiency decreased as the inclination increased; and when the inclination was greater than 25°, the oil/water separation efficiency decreased significantly as the inclination increased.

### Oil and water volume phase distributions

**Fig 3:**
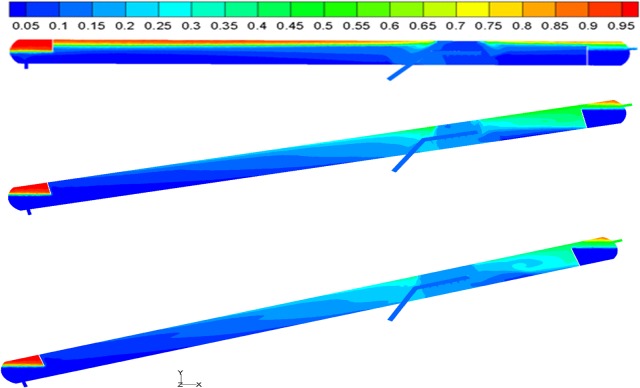
Oil and water volume distributions at different inclinations. (A) Inclination = 0°; (B) Inclination = 12° and (C) Inclination = 18°.

Through numerical simulations, the distributions of oil and water in the separator were obtained. The volume distributions of oil in the axial sections for some of the calculation models are shown in **Fig 3**.

As shown in **Fig 3**, the two phases near the separator dispenser mixed vigorously, and the isoline of the oil/water interface exhibited obvious bends. In addition, as the inclination increased, the “dead oil” and “dead water” within the separator reduced gradually; the inclined oil/water separator exhibited a significant reduction in “dead oil” and “dead water” compared to the conventional horizontal oil/water separator. Furthermore, as the inclination increased, the separator’s oil/water interface fluctuated greatly; when the inclination increased to 18°, the oil/water interface fluctuated considerably, and “swirling flow” occurred in the upper region. The fluctuations in the oil/water interface could cause the separated oil and water phases at the interface to mix.

### Droplet trajectories

**Fig 4:**
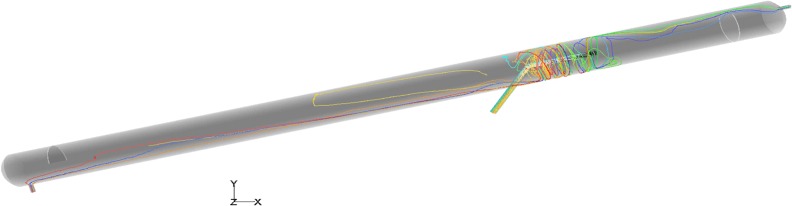
The droplet trajectories within a separator with a length/diameter ratio of 15.

**Fig 5:**
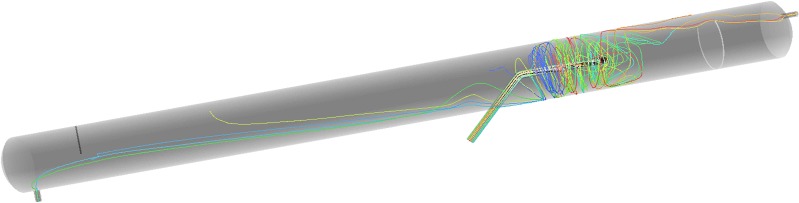
The droplet trajectories within a separator with a length/diameter ratio of 10.

The effect of flow fields on droplet motion depends on the trajectories of those droplets. The droplet trajectories within the oil/water separator at an inclination of 12° were obtained through numerical simulations, as shown in **Fig 4**. In addition, a separator with a length/diameter ratio of 10 was simulated in order to study the effect of the length/diameter ratio on droplet trajectories, as shown in **Fig 5**.

According to **Fig 4 and Fig 5**, when the length/diameter ratio was equal to 10, some of the oil droplets that entered the separator were not separated, and the swirling flow structure of the liquid droplets at the dispenser was complex. When the length/diameter ratio was equal to 15, only a small portion of the oil droplets that entered the separator were not separated, and the separation effect was satisfactory. Thus, a separator with a length/diameter ratio of 15 and a 12° inclination could meet the requirements and obtain a desired oil/water separation effect.

### Velocity field distribution

**Fig 6:**
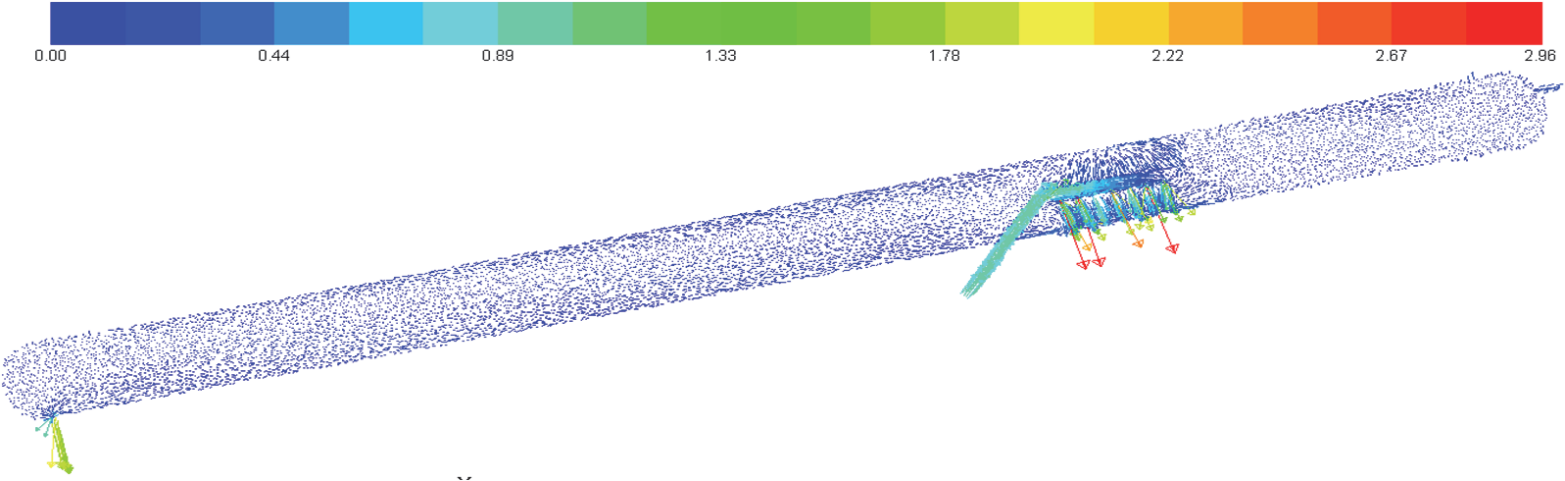
The velocity vectors for a 12° inclination.

The velocity vectors within the inclined oil/water separator are shown in **Fig 6**.

According to **Fig 6**, after flowing through the dispenser, the oil and water phases sprayed onto the lower wall of the dispenser at high rates. After the impact, swirls were generated between the dispenser and the wall of the separation tank so that the oil and water phases passed through the dispenser region at high speeds. Mild swirls were also generated after collision with the weir.

## Structural Optimization

A large number of structural parameters affect the separation performance of an inclined oil/water separator, and any structural change could affect the efficiency of the separator. In order to prevent complicated experiments and improve efficiency without compromising applicability, orthogonal array experiments were used to study the effects of the main structural parameters on the oil/water separation efficiency. Through horizontal comparison, the optimal structural parameters for the inclined oil/water separator were obtained.

### Orthogonal array experiments

#### (1) Influencing factors and their levels

An inclined oil/water separator primarily consists of the separation tank, dispenser, oil outlet, water outlet, oil weir, and water weir. In this study, the effects of eleven factors, including the weir height, weir position, hole diameter and spacing of the dispenser, horizontal and longitudinal positions of the dispenser, and the structure of the oil outlet, on separation efficiency were analyzed, and an orthogonal array at five levels, whose factors and levels are shown in **S2 Table**, was designed [14–16].

This experimental design consisted of eleven factors with five levels for each factor. There could be 5^11^ experimental conditions for a comprehensive experimental design; however, this would be impractical. Thus, a *L*_50_(5^11^) orthogonal array was selected, which consisted of fifty simulations (**S3 Table**).

#### (2) Analysis of the results

Fifty simulations were performed for eleven factors at five levels. The averages and ranges of the separation efficiencies for the various factors at different levels are shown in **S4 Table**.

The significance of the various structural parameters on the oil/water separation efficiency was determined through a range analysis of the numerical simulation results. A large range indicated that the oil/water separation efficiencies for a certain factor at different levels were significantly different and that the factor had a significant effect on the oil/water separation efficiency. A small range indicated that this factor had a small effect on the oil/water separation efficiency. The significance levels of the various factors on the oil/water separation efficiency in descending order are shown in **Table 3**. (Note: The higher the significance level number, the less important the corresponding influencing factor is.)

**Table 3:** Range analysis.

Order of significance levels	Influencing factors	Range
1	horizontal position of the dispenser	0.111
2	hole number of the dispenser	0.069
3	hole diameter of the dispenser	0.068
4	oil weir height	0.068
5	water weir height	0.067
6	oil weir position	0.063
7	separator inclination	0.055
8	oil outlet position	0.051
9	hole spacing of the dispenser	0.046
10	longitudinal position of the dispenser	0.036
11	water weir position	0.020

According to **Table 3**, the horizontal position, hole number, and hole diameter of the dispenser had significant effects on the oil/water separation efficiency; the longitudinal position of the dispenser and the position of the water weir had insignificant effects on the oil/water separation efficiency; and the other factors had effects that fell between those extremities.

#### (3) Effects of the structural parameters on separation efficiency

The changes in the factor levels had significant effects on the oil/water separation efficiency; the study of these effects is of great importance in separator optimization. Numerical simulations were performed under various conditions, such as different inclination and oil weir parameters; only one variable was used in each simulation. For example, in the analysis of the effect of inclination on separation efficiency, the inclination alone was changed. The obtained optimal parameter values are shown in **S5 Table** (Note: X represent separation efficiency).

### Structural optimization

**Fig 7:**
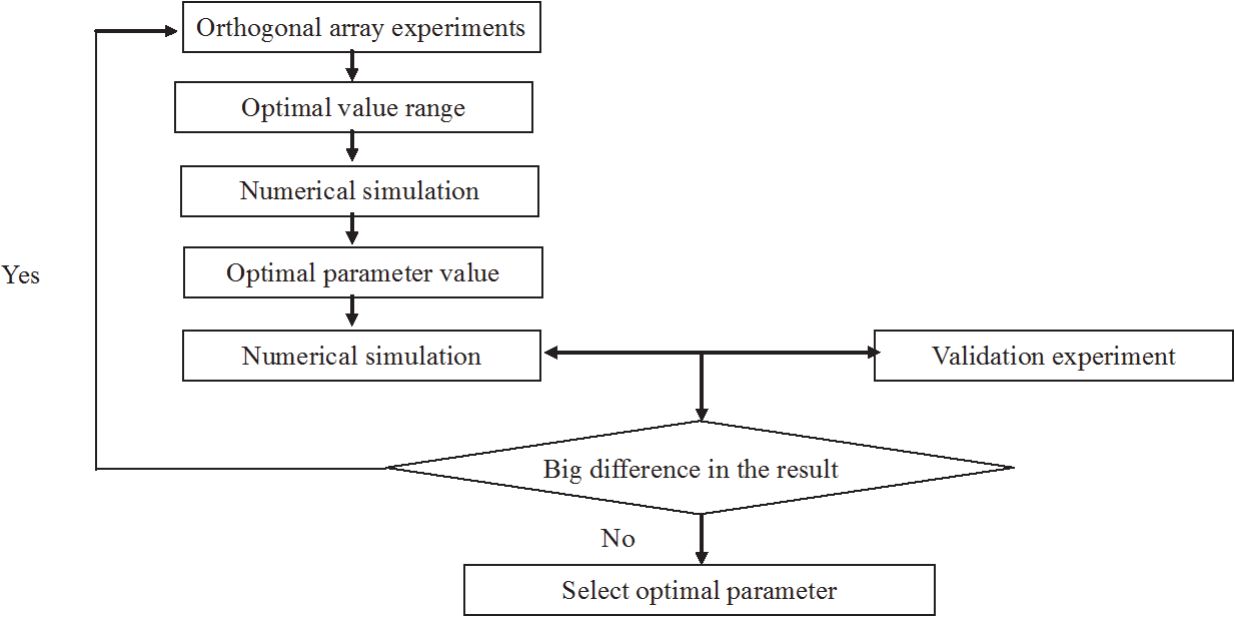
The block flow diagram for selecting optimal parameters.

In order to obtain the optimal structural parameters of the inclined oil/water separator, the factor levels that demonstrated the highest separation efficiencies were combined with the structural parameters that demonstrated the highest separation efficiencies, as shown in **Fig 7**. The results are shown in **Table 4**.

**Table 4:** Optimal structural parameters obtained through computational analysis.

Inclination	Oil weir height	Water weir height	Oil weir position	Water weir position	Dispenser					Oil outlet position
					Hole diameter	Hole spacing	Hole number	Horizontal position	Longitudinal position	
12°	0.8D	0.4D	0.5D	2.0D	2.0cm	3.0d	8	0.80L_e_	0.5D	0.8D

The model with the optimal structural parameters obtained through computational analysis was not included in the orthogonal array of fifty experiments. Since the fifty simulations comprised only a fraction of the comprehensive experiments, a validation experiment was necessary to analyze other possible optimal structural parameters and examine the reproducibility of the optimal structural parameters. In addition, the results had to be compared to the results of the fifty simulations in order to determine the quality of the optimal structural parameters obtained through computational analysis.

A numerical simulation was executed for the inclined oil/water separator shown in **Table 4**under the same conditions mentioned above. The result was compared to the first three simulation results obtained through range analysis in order to determine the optimal structural parameters of the inclined oil/water separator, as shown in **Table 5**.

**Table 5:** A comparison between the result of the validation experiment and the first three groups of the simulation results.

Experiment No.		Water content at the oil outlet	Oil content at the water outlet	Oil/water separation efficiency
Range analysis	Experiment 4	44.31%	0.50%	95.0%
	Experiment 14	79.15%	0.46%	95.4%
	Experiment 31	49.36%	0.50%	95.0%
Computational analysis	Validation experiment	39.67%	0.50%	95.0%

According to the validation experiment, the separation efficiency for the optimal structural parameters obtained through the computational model was 95.0%, which was lower than that of the optimal structural parameters obtained through range analysis. This was primarily due to the errors or incomplete factors considered in the simulations. However, the difference in separation efficiency was found to be insignificant, indicating that the fifty simulations were typical. The water content of the oil outlet in the model obtained through computational analysis was only 39.67% but was as high as 79.15% in the model obtained through range analysis. Despite the insignificant difference in separation efficiency, the difference in the water content of the oil outlet was significant. Due to the water content of the oil outlet and the oil content of the water outlet, the optimal structural parameters obtained through computational analysis was selected as the optimal structural model of the inclined oil/water separator simulated in this study.

The comparison of the separation efficiency of the inclined oil/water separator before and after structural parameter optimization is shown in **Table 6**.

**Table 6:** Comparison of the separation efficiencies before and after optimization of structural parameters.

Item	Water content at the oil outlet	Oil content at the water outlet	Separation efficiency
Before the optimization of structural parameters	57.50%	0.95%	90.48%
After the optimization of structural parameters	39.67%	0.50%	95.0%

As shown in **Table 6**, after optimization of the original structural parameters of the inclined oil/water separator, the oil/water separation efficiency improved by 4.996%, and the oil outlet water content decreased from 57.50% to 39.67%.

**Fig 8:**
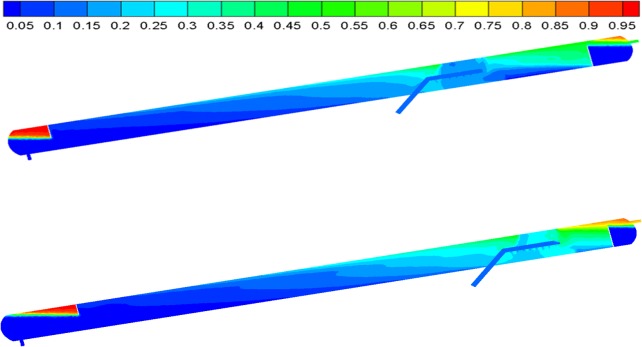
Oil volume distributions. (A) Before optimization and (B) After optimization.

The flow field within the inclined oil/water separator with optimized structural parameters was analyzed in order to study the distribution. The oil and water volume distributions within the separator before and after structural parameter optimization are shown in **Fig 8**.

As indicated by the oil and water volume distributions before and after structural parameter optimization, the “dead oil” and “dead water” volumes within the separator decreased significantly at a 12° inclination. In addition, the area affected by swirls in the region between the dispenser and the oil weir decreased, and the swirl intensity decreased significantly after structural parameter optimization. Furthermore, the oil volume content of the oil outlet increased significantly.

According to this information, the separation performance of the inclined oil/water separator under equal conditions improved after structural parameter optimization.

## Field Experiment

**Fig 9:**
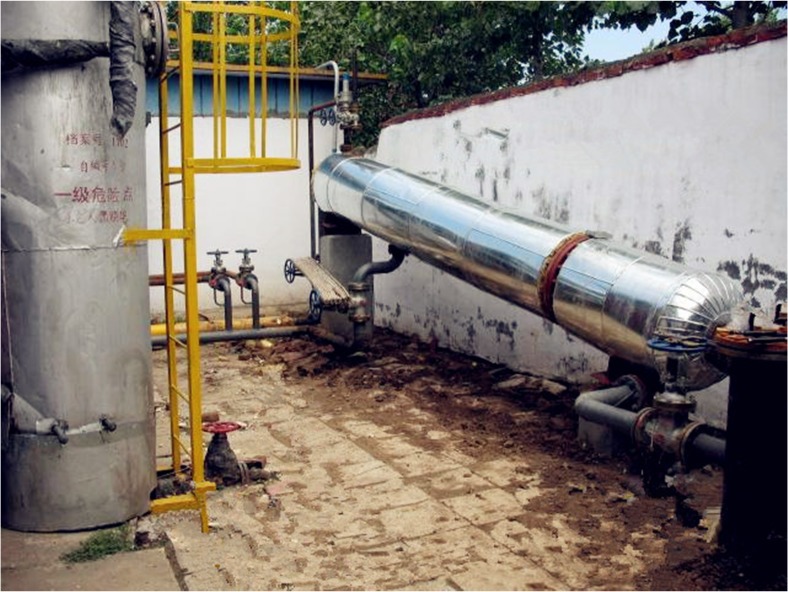
The field experiment on the inclined separator.

In order to validate the accuracy of the numerical simulation and validate the improved separation resulting from structural parameter optimization, a field experiment was performed for comparison. An inclined oil/water separation device was manufactured based on the optimized model parameters (**Fig 9**), installed at Metering Station 20# in Block XX of an oilfield, and compared to the inclined oil/water separator without structural optimization used in Metering Station 15# in the same block.

### Experimental scheme

Many factors affect oil/water separation efficiency. Due to the limitations of the objective conditions, conducting experiments on the effects of numerous structural parameters on oil/water separation efficiency would have been inconvenient. Therefore, in order to validate the accuracy of the simulation results, the field experiment was only used to study the effects of inclination and residence time on oil/water separation efficiency.

A field experiment was conducted to investigate the oil/water separation of the liquid produced by Oil Production Crew Twelve at inclinations ranging from approximately 0° to 15° with a settling time of 6 min. In order to analyze the effect of inclination on oil/water separation efficiency, the experimental results were compared to the numerical simulation results as well as the results of the separation experiment of the inclined oil/water separator used at Metering Station 15#. After the optimal inclination was determined, the amount of liquid used in the separator was altered in order to study the effect of residence time on oil/water separation efficiency and indicate the optimal residence time for field applications.

Samples were obtained from the liquid inlet, oil outlet, and water outlet every two hours for each experiment; three samples were obtained from each source. The water contents of the three groups of samples were measured and averaged. The separation efficiency was calculated for each separator. The water contents of the samples were represented by volumetric water content for comparison with the numerical simulation results.

### Analysis of the experimental results

#### (1) Inclination-separation efficiency

**Fig 10:**
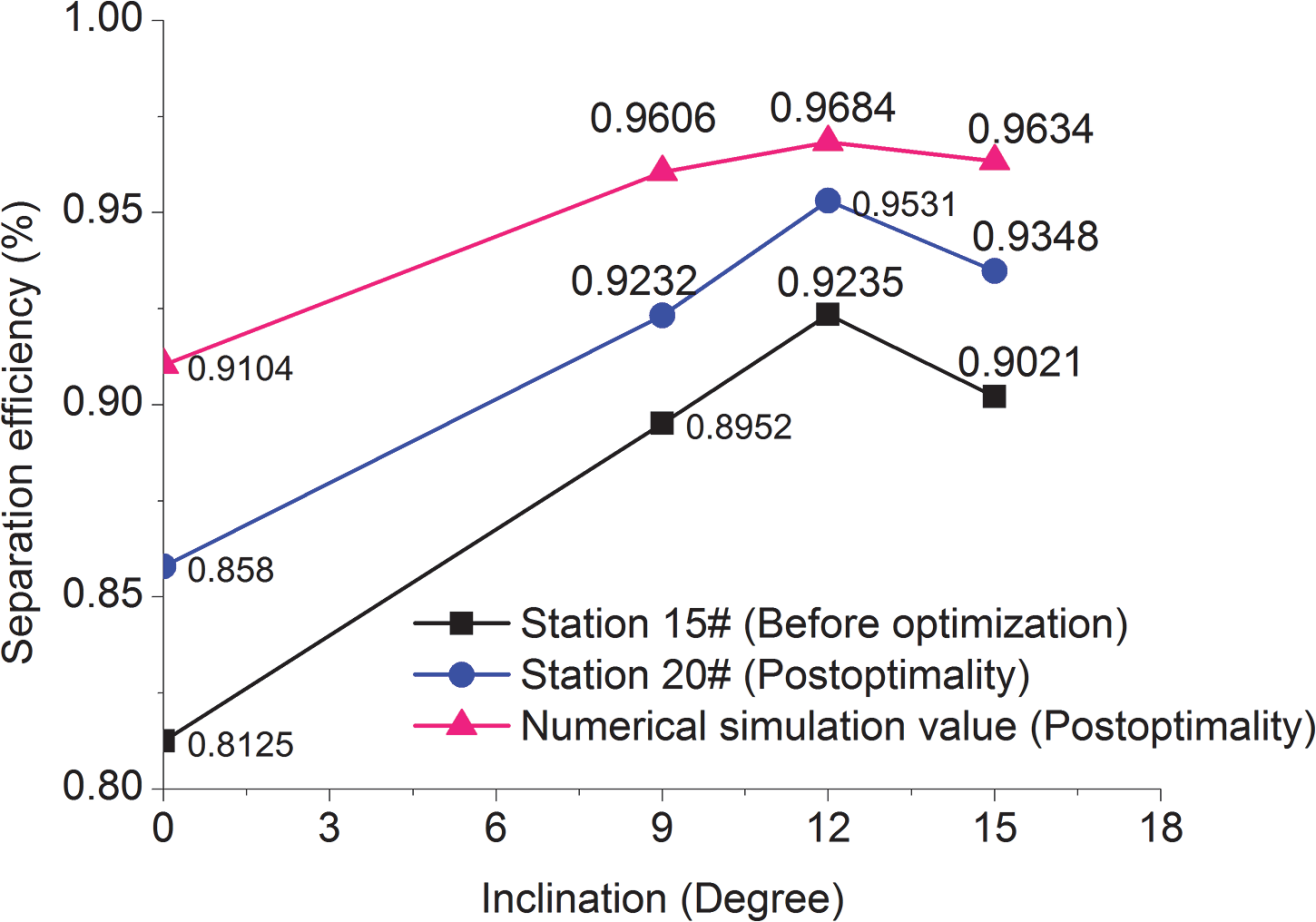
Inclination-separation efficiency.

Based on the numerical simulation results, a field experiment was conducted on the inclined oil/water separator at inclinations of 0°, 9°, 12°, and 15° at the Metering Station. The changes in inclination yielded the oil/water separation efficiencies shown in **Fig 10 (S6 Table)**.

According to **Fig 10**, the oil/water separation efficiency increased as the inclination increased for the same incoming liquid residence time. When the inclination ranged between approximately 9° and 15°, the oil/water separation efficiency changed slightly; when the inclination was equal to 12°, the oil/water separation was most effective, with a separation efficiency of 96.84%.

When the inclination ranged from 0° to 15°, the separation efficiency of the inclined oil/water separator with optimized structural parameters improved under equal conditions. When the inclination was equal to 12°, the separation efficiency increased by 4.64%, or from 92.35% to 96.84%.

#### (2) Residence time-separation efficiency

Residence time has an important effect on separation efficiency. When selecting the residence time, various factors should be considered. A shorter residence time is desirable as it satisfies the separation requirement. In order to study the effect of residence time on the separation efficiency of the inclined oil/water separator, the inclination was maintained at 12°, and the amount of liquid in the separator was altered in the field experiment.

**Fig 11:**
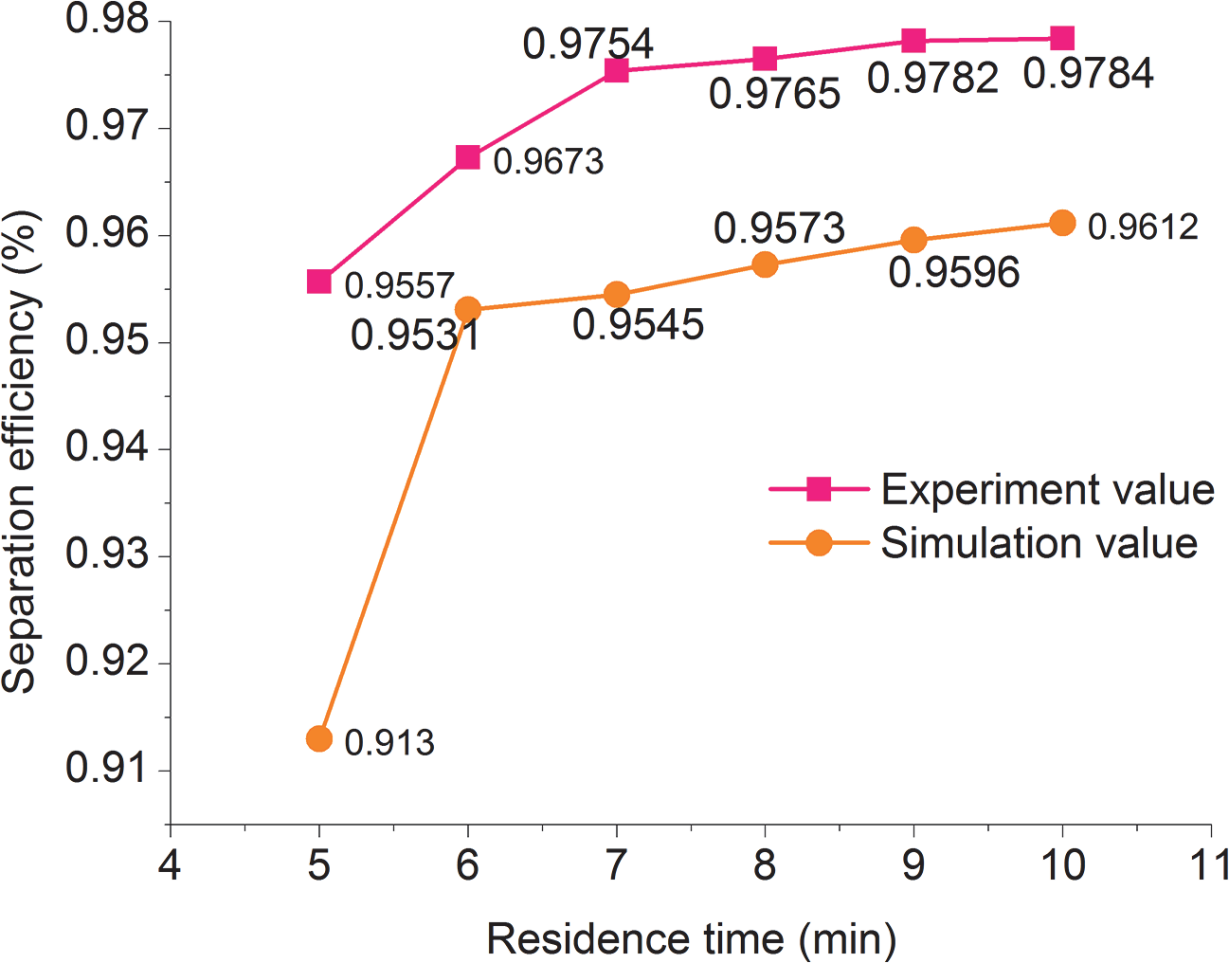
Residence time-separation efficiency.

According to **Fig 11 (S7 Table)**, as the residence time increased, the oil/water separation efficiency increased rapidly. When the residence time reached 7 min, the oil/water separation efficiency increased rapidly to greater than 97%, and the content of the water outlet decreased to 0.23%. As the residence time increased to greater than 7 min, the oil/water separation efficiency increased slightly. Therefore, a further increase in residence time would have an insignificant effect on the improvement of oil/water separation efficiency.

According to **Fig 10 and Fig 11**, there were minor errors in the experimental and simulation data, but the changes in the separation efficiency resulting from the variations in inclination and residence time were basically consistent. This indicated the feasibility of the optimization of the structural parameters of the oil/water separator and the prediction of its separation efficiency through numerical simulations indicating that the structural parameter optimization of the oil/water separator.

## Conclusions

In this paper, the inclined oil/water separator, which is used to improve the oil/water separation efficiency for oilfields at the high water cut stage, was studied. Numerical simulations were executed in order to analyze the distribution of oil and water in the separator and the effects of structural parameters on oil/water separation performance. Orthogonal array experiments were conducted to study the effects of structural parameters on oil/water separation efficiency, and optimal structural parameter values were obtained. The numerical simulations were validated through a field experiment, and, thus, a basis for the design and widespread application of this type of separator was provided. Through these experiments, the following conclusions were drawn.

1. A direct understanding of the oil and water distribution and flow field within the separator was obtained through numerical simulations. The separation efficiency of the inclined separator was higher than that of the conventional horizontal separator under the same conditions.
2. The structural parameters had different effects on the oil/water separation efficiency. The horizontal position, hole number, and hole diameter of the dispenser had significant effects on the separation efficiency, whereas the longitudinal position of the dispenser and the water weir position had insignificant effects on the separation efficiency.
3. Orthogonal array experiments were conducted to study the effects of the structural parameters on the oil/water separation efficiency, and optimal structural parameters were obtained. After optimization of the structural parameters, the oil/water separation efficiency increased by 4.996% to a value of 95%.
4. A field experiment was conducted on the inclined oil/water separator at Metering Station 20# in Block XX of an oilfield. The results indicated that the inclined oil/water separator had a high handling capacity and a significant separation effect. After comparing these results to those of the numerical simulation, some differences were found, but the effects of the structural parameters on the separation efficiency were consistent in both data sets. Therefore, optimizing the structure of the oil/water separator and predicting its separation efficiency would be feasible.

## Supporting Information

S1 Table Statistics concerning the separation characteristics at different inclinations. (DOC)

S2 Table An array of the influential factors and their levels. (DOC)

S3 Table Orthogonal experiments and results. (DOC)

S4 Table The statistics concerning the results of the range analysis. (DOC)

S5 Table Optimal values for the parameters. (DOC)

S6 Table The effect of inclination on oil/water separation efficiency. (DOC)

S7 Table The effect of residence time on oil/water separation efficiency. (DOC)
