# Supplementary material for: Numerical Simulation and Structural Optimization of the Inclined Oil/Water Separator
Source: PLoS One. 2015 Apr 13;10(4):e0124095. doi: 10.1371/journal.pone.0124095 (PMC4395151; doi:10.1371/journal.pone.0124095)
Supplement: S1 Table — (DOC) [file pone.0124095.s001.doc]

**Table S1: Statistics concerning the separation characteristics at different inclinations**

| Degree | Water content at the oil outlet | Oil content at the water outlet | Separation efficiency |
| --- | --- | --- | --- |
| 0° | 82.83% | 2.50% | 75.00% |
| 3° | 63.95% | 2.22% | 77.77% |
| 6° | 54.69% | 2.15% | 78.54% |
| 9° | 71.16% | 1.41% | 85.89% |
| 12° | 57.50% | 0.95% | 90.48% |
| 15° | 49.83% | 1.50% | 85.00% |
| 18° | 50.11% | 1.39% | 86.14% |
| 21° | 57.69% | 1.41% | 85.95% |
| 25° | 57.33% | 1.50% | 85.00% |
| 30° | 52.72% | 2.33% | 76.75% |
