# Supplementary material for: Numerical Simulation and Structural Optimization of the Inclined Oil/Water Separator
Source: PLoS One. 2015 Apr 13;10(4):e0124095. doi: 10.1371/journal.pone.0124095 (PMC4395151; doi:10.1371/journal.pone.0124095)
Supplement: S2 Table — (DOC) [file pone.0124095.s002.doc]

**Table S2: An array of the influential factors and their levels**

| Levels | Inclination | Oil weir height | Water weir height | Oil weir position | Water weir position | Dispenser | | | | | Oil outlet position |
| --- | --- | --- | --- | --- | --- | --- | --- | --- | --- | --- | --- |
|  |  |  |  |  |  | Hole diameter | Hole spacing | Hole number | Horizontal position | Longitudinal position |  |
| Level 1 | 6° | 0.5D | 0.4D | 0.5D | 0.5D | 1.0cm | 2.0d | 5 | 50%Le | 0.2D | bottom |
| Level 2 | 9° | 0.6D | 0.5D | 1.0D | 1.0D | 1.5cm | 2.5d | 6 | 60%Le | 0.4D | top |
| Level 3 | 12° | 0.7D | 0.6D | 1.5D | 1.5D | 2.0cm | 3.0d | 7 | 70%Le | 0.5D | 0.6D |
| Level 4 | 15° | 0.8D | 0.7D | 2.0D | 2.0D | 2.5cm | 3.5d | 8 | 75%Le | 0.6D | 0.7D |
| Level 5 | 18° | 0.9D | 0.8D | 3.0D | 3.0D | 3.0cm | 4.0d | 9 | 80%Le | 0.8D | 0.8D |
