# Supplementary material for: Numerical Simulation and Structural Optimization of the Inclined Oil/Water Separator
Source: PLoS One. 2015 Apr 13;10(4):e0124095. doi: 10.1371/journal.pone.0124095 (PMC4395151; doi:10.1371/journal.pone.0124095)
Supplement: S3 Table — (DOC) [file pone.0124095.s003.doc]

**Table S3: Orthogonal experiments and results**

| Orthogonal experiments | Inclination | Height | | Horizontal position | | Dispenser | | | | | oil outlet position | Oil content at the water outlet | Water content at the oil outlet | Separation efficiency |
| --- | --- | --- | --- | --- | --- | --- | --- | --- | --- | --- | --- | --- | --- | --- |
|  |  | Oil weir | Water weir | Oil weir | Water weir | Hole diameter | Hole spacing | Hole number | Horizontal position | Longitudinal position |  |  |  |  |
| Experiment 1 | 6° | 0.5D | 0.4D | 0.5D | 0.5D | 1.0cm | 2.0d | 5 | 50%Le | 0.2D | Bottom | 59.66% | 2.07% | 79.27% |
| Experiment 2 | 6° | 0.6D | 0.5D | 1.0D | 1.0D | 1.5cm | 2.5d | 6 | 60%Le | 0.4D | Top | 54.62% | 1.87% | 81.33% |
| Experiment 3 | 6° | 0.7D | 0.6D | 1.5D | 1.5D | 2.0cm | 3.0d | 7 | 70%Le | 0.5D | 0.6D | 60.66% | 0.92% | 90.78% |
| Experiment 4 | 6° | 0.8D | 0.7D | 2.0D | 2.0D | 2.5cm | 3.5d | 8 | 75%Le | 0.6D | 0.7D | 44.31% | 0.50% | 95.00% |
| Experiment 5 | 6° | 0.9D | 0.8D | 3.0D | 3.0D | 3.0cm | 4.0d | 9 | 80%Le | 0.8D | 0.8D | 54.56% | 1.41% | 85.95% |
| Experiment 6 | 9° | 0.5D | 0.5D | 1.5D | 2.0D | 3.0cm | 2.0d | 6 | 70%Le | 0.6D | 0.8D | 33.31% | 1.41% | 85.95% |
| Experiment 7 | 9° | 0.6D | 0.6D | 2.0D | 3.0D | 1.0cm | 2.5d | 7 | 75%Le | 0.8D | Bottom | 92.01% | 4.86% | 51.40% |
| Experiment 8 | 9° | 0.7D | 0.7D | 3.0D | 0.5D | 1.5cm | 3.0d | 8 | 80%Le | 0.2D | Top | 48.27% | 1.41% | 85.89% |
| Experiment 9 | 9° | 0.8D | 0.8D | 0.5D | 1.0D | 2.0cm | 3.5d | 9 | 50%Le | 0.4D | 0.6D | 46.44% | 2.36% | 76.44% |
| Experiment 10 | 9° | 0.9D | 0.4D | 1.0D | 1.5D | 2.5cm | 4.0d | 5 | 60%Le | 0.5D | 0.7D | 57.71% | 1.40% | 86.01% |
| Experiment 11 | 12° | 0.5D | 0.6D | 3.0D | 1.0D | 2.5cm | 3.5d | 5 | 70%Le | 0.8D | Top | 47.50% | 1.50% | 84.98% |
| Experiment 12 | 12° | 0.6D | 0.7D | 0.5D | 1.5D | 3.0cm | 4.0d | 6 | 75%Le | 0.2D | 0.6D | 20.75% | 0.79% | 92.06% |
| Experiment 13 | 12° | 0.7D | 0.8D | 1.0D | 2.0D | 1.0cm | 2.0d | 7 | 80%Le | 0.4D | 0.7D | 78.54% | 1.41% | 85.90% |
| Experiment 14 | 12° | 0.8D | 0.4D | 1.5D | 3.0D | 1.5cm | 2.5d | 8 | 50%Le | 0.5D | 0.8D | 79.15% | 0.46% | 95.44% |
| Experiment 15 | 12° | 0.9D | 0.5D | 2.0D | 0.5D | 2.0cm | 3.0d | 9 | 60%Le | 0.6D | Bottom | 53.62% | 1.41% | 85.90% |
| Experiment 16 | 15° | 0.5D | 0.7D | 1.0D | 3.0D | 2.0cm | 4.0d | 7 | 50%Le | 0.6D | Top | 50.65% | 1.93% | 80.71% |
| Experiment 17 | 15° | 0.6D | 0.8D | 1.5D | 0.5D | 2.5cm | 2.0d | 8 | 60%Le | 0.8D | 0.6D | 34.82% | 1.53% | 84.66% |
| Experiment 18 | 15° | 0.7D | 0.4D | 2.0D | 1.0D | 3.0cm | 2.5d | 9 | 70%Le | 0.2D | 0.7D | 26.98% | 1.36% | 86.45% |
| Experiment 19 | 15° | 0.8D | 0.5D | 3.0D | 1.5D | 1.0cm | 3.0d | 5 | 75%Le | 0.4D | 0.8D | 61.86% | 1.41% | 85.88% |
| Experiment 20 | 15° | 0.9D | 0.6D | 0.5D | 2.0D | 1.5cm | 3.5d | 6 | 80%Le | 0.5D | Bottom | 79.66% | 0.56% | 94.40% |
| Experiment 21 | 18° | 0.5D | 0.8D | 2.0D | 1.5D | 1.5cm | 3.5d | 7 | 60%Le | 0.2D | 0.8D | 38.20% | 2.40% | 76.04% |
| Experiment 22 | 18° | 0.6D | 0.4D | 3.0D | 2.0D | 2.0cm | 4.0d | 8 | 70%Le | 0.4D | Bottom | 43.85% | 1.45% | 85.52% |
| Experiment 23 | 18° | 0.7D | 0.5D | 0.5D | 3.0D | 2.5cm | 2.0d | 9 | 75%Le | 0.5D | Top | 35.63% | 1.39% | 86.11% |
| Experiment 24 | 18° | 0.8D | 0.6D | 1.0D | 0.5D | 3.0cm | 2.5d | 5 | 80%Le | 0.6D | 0.6D | 43.02% | 1.39% | 86.06% |
| Experiment 25 | 18° | 0.9D | 0.7D | 1.5D | 1.0D | 1.0cm | 3.0d | 6 | 50%Le | 0.8D | 0.7D | 50.33% | 2.50% | 75.00% |
| Experiment 26 | 6° | 0.5D | 0.4D | 2.0D | 3.0D | 2.5cm | 3.0d | 6 | 80%Le | 0.4D | 0.6D | 34.27% | 0.99% | 90.09% |
| Experiment 27 | 6° | 0.6D | 0.5D | 3.0D | 0.5D | 3.0cm | 3.5d | 7 | 50%Le | 0.5D | 0.7D | 42.37% | 2.63% | 73.73% |
| Experiment 28 | 6° | 0.7D | 0.6D | 0.5D | 1.0D | 1.0cm | 4.0d | 8 | 60%Le | 0.6D | 0.8D | 54.94% | 1.39% | 86.06% |
| Experiment 29 | 6° | 0.8D | 0.7D | 1.0D | 1.5D | 1.5cm | 2.0d | 9 | 70%Le | 0.8D | Bottom | 66.80% | 0.94% | 90.56% |
| Experiment 30 | 6° | 0.9D | 0.8D | 1.5D | 2.0D | 2.0cm | 2.5d | 5 | 75%Le | 0.2D | Top | 49.38% | 1.41% | 85.85% |
| Experiment 31 | 9° | 0.5D | 0.5D | 0.5D | 1.5D | 2.0cm | 2.5d | 8 | 80%Le | 0.8D | 0.7D | 49.36% | 0.50% | 95.0% |
| Experiment 32 | 9° | 0.6D | 0.6D | 1.0D | 2.0D | 2.5cm | 3.0d | 9 | 50%Le | 0.2D | 0.8D | 38.87% | 2.18% | 78.17% |
| Experiment 33 | 9° | 0.7D | 0.7D | 1.5D | 3.0D | 3.0cm | 3.5d | 5 | 60%Le | 0.4D | Bottom | 45.25% | 1.41% | 85.85% |
| Experiment 34 | 9° | 0.8D | 0.8D | 2.0D | 0.5D | 1.0cm | 4.0d | 6 | 70%Le | 0.5D | Top | 58.45% | 1.71% | 82.88% |
| Experiment 35 | 9° | 0.9D | 0.4D | 3.0D | 1.0D | 1.5cm | 2.0d | 7 | 75%Le | 0.6D | 0.6D | 76.61% | 1.26% | 87.44% |
| Experiment 36 | 12° | 0.5D | 0.6D | 1.5D | 0.5D | 1.5cm | 4.0d | 9 | 75%Le | 0.4D | 0.7D | 62.55% | 1.38% | 86.19% |
| Experiment 37 | 12° | 0.6D | 0.7D | 2.0D | 1.0D | 2.0cm | 2.0d | 5 | 80%Le | 0.5D | 0.8D | 51.87% | 0.93% | 90.68% |
| Experiment 38 | 12° | 0.7D | 0.8D | 3.0D | 1.5D | 2.5cm | 2.5d | 6 | 50%Le | 0.6D | Bottom | 62.17% | 3.50% | 65.00% |
| Experiment 39 | 12° | 0.8D | 0.4D | 0.5D | 2.0D | 3.0cm | 3.0d | 7 | 60%Le | 0.8D | Top | 39.10% | 0.71% | 92.88% |
| Experiment 40 | 12° | 0.9D | 0.5D | 1.0D | 3.0D | 1.0cm | 3.5d | 8 | 70%Le | 0.2D | 0.6D | 71.04% | 0.94% | 90.61% |
| Experiment 41 | 15° | 0.5D | 0.7D | 3.0D | 2.0D | 1.0cm | 2.5d | 9 | 60%Le | 0.5D | 0.6D | 42.34% | 2.18% | 78.18% |
| Experiment 42 | 15° | 0.6D | 0.8D | 0.5D | 3.0D | 1.5cm | 3.0d | 5 | 70%Le | 0.6D | 0.7D | 71.54% | 1.39% | 86.08% |
| Experiment 43 | 15° | 0.7D | 0.4D | 1.0D | 0.5D | 2.0cm | 3.5d | 6 | 75%Le | 0.8D | 0.8D | 52.83% | 0.83% | 91.67% |
| Experiment 44 | 15° | 0.8D | 0.5D | 1.5D | 1.0D | 2.5cm | 4.0d | 7 | 80%Le | 0.2D | Bottom | 28.08% | 1.28% | 87.18% |
| Experiment 45 | 15° | 0.9D | 0.6D | 2.0D | 1.5D | 3.0cm | 2.0d | 8 | 50%Le | 0.4D | Top | 47.90% | 2.37% | 76.32% |
| Experiment 46 | 18° | 0.5D | 0.8D | 1.0D | 1.0D | 3.0cm | 3.0d | 8 | 75%Le | 0.5D | Bottom | 64.18% | 1.47% | 85.34% |
| Experiment 47 | 18° | 0.6D | 0.4D | 1.5D | 1.5D | 1.0cm | 3.5d | 9 | 80%Le | 0.6D | Top | 71.99% | 1.38% | 86.22% |
| Experiment 48 | 18° | 0.7D | 0.5D | 2.0D | 2.0D | 1.5cm | 4.0d | 5 | 50%Le | 0.8D | 0.6D | 43.97% | 2.38% | 76.19% |
| Experiment 49 | 18° | 0.8D | 0.6D | 3.0D | 3.0D | 2.0cm | 2.0d | 6 | 60%Le | 0.2D | 0.7D | 48.12% | 1.39% | 86.06% |
| Experiment 50 | 18° | 0.9D | 0.7D | 0.5D | 0.5D | 2.5cm | 2.5d | 7 | 70%Le | 0.4D | 0.8D | 37.40% | 1.47% | 85.29% |
