# Supplementary material for: Numerical Simulation and Structural Optimization of the Inclined Oil/Water Separator
Source: PLoS One. 2015 Apr 13;10(4):e0124095. doi: 10.1371/journal.pone.0124095 (PMC4395151; doi:10.1371/journal.pone.0124095)
Supplement: S4 Table — (DOC) [file pone.0124095.s004.doc]

**Table S4: The statistics concerning the results of the range analysis**

| Levels | Inclination | Oil weir height | Water weir height | Oil weir position | Water weir position | Dispenser | | | | | Oil outlet position |
| --- | --- | --- | --- | --- | --- | --- | --- | --- | --- | --- | --- |
|  |  |  |  |  |  | Hole diameter | Hole spacing | Hole number | Horizontal position | Longitudinal position |  |
| Level 1 | 0.859 | 0.842 | 0.881 | 0.874 | 0.842 | 0.801 | 0.853 | 0.847 | 0.776 | 0.848 | 0.810 |
| Level 2 | 0.815 | 0.810 | 0.848 | 0.856 | 0.841 | 0.860 | 0.810 | 0.844 | 0.843 | 0.839 | 0.843 |
| Level 3 | 0.870 | 0.840 | 0.820 | 0.863 | 0.844 | 0.869 | 0.856 | 0.811 | 0.869 | 0.864 | 0.853 |
| Level 4 | 0.852 | 0.878 | 0.859 | 0.811 | 0.858 | 0.842 | 0.855 | 0.880 | 0.847 | 0.844 | 0.855 |
| Level 5 | 0.828 | 0.853 | 0.814 | 0.819 | 0.838 | 0.851 | 0.849 | 0.840 | 0.887 | 0.828 | 0.861 |
| Range | 0.055 | 0.068 | 0.067 | 0.063 | 0.020 | 0.068 | 0.046 | 0.069 | 0.111 | 0.036 | 0.051 |
