# Supplementary material for: Numerical Simulation and Structural Optimization of the Inclined Oil/Water Separator
Source: PLoS One. 2015 Apr 13;10(4):e0124095. doi: 10.1371/journal.pone.0124095 (PMC4395151; doi:10.1371/journal.pone.0124095)
Supplement: S5 Table — (DOC) [file pone.0124095.s005.doc]

**Table S5: Optimal values for the parameters**

| Structural parameters | Inclination-separation efficiency | | Structural parameters of the oil weir and separation efficiency | | | | Structural parameters of the water weir and separation efficiency | | | | Structural parameters of the dispenser and separation efficiency | | | | | | | | | | Oil outlet position-separation efficiency | |
| --- | --- | --- | --- | --- | --- | --- | --- | --- | --- | --- | --- | --- | --- | --- | --- | --- | --- | --- | --- | --- | --- | --- |
|  |  |  | height-separation efficiency | | position-separation efficiency | | height-separation efficiency | | position-separation efficiency | | Hole diameter-separation efficiency | | Hole spacing-separation efficiency | | Hole number-separation efficiency | | Horizontal position-separation efficiency | | Longitudinal position-separation efficiency | |  |  |
| No. | Values | X (%) | Values | X (%) | Values | X (%) | Values | X (%) | Values | X (%) | Values | X (%) | Values | X (%) | Values | X (%) | Values | X (%) | Values | X (%) | Values | X (%) |
| 1 | 6° | 85.9 | 0.5D | 84.2 | 0.5D | 87.4 | 0.4D | 88.1 | 0.5D | 84.2 | 1cm | 80.1 | 2.0d | 85.3 | 5 | 84.7 | 0.5Le | 77.6 | 0.2D | 84.8 | bottom | 81.0 |
| 2 | 9° | 81.5 | 0.6D | 81.0 | 1.0D | 85.6 | 0.5D | 84.8 | 1.0D | 84.1 | 1.5cm | 86.0 | 2.5d | 81.0 | 6 | 84.4 | 0.6Le | 84.3 | 0.4D | 83.9 | 0.6D | 85.3 |
| 3 | 12° | 87.0 | 0.7D | 84.0 | 1.5D | 86.3 | 0.6D | 82.0 | 1.5D | 84.4 | 2cm | 86.9 | 3.0d | 85.6 | 7 | 81.1 | 0.7Le | 86.9 | 0.5D | 86.4 | 0.7D | 85.5 |
| 4 | 15° | 85.2 | 0.8D | 87.8 | 2.0D | 81.1 | 0.7D | 85.9 | 2.0D | 85.8 | 2.5cm | 84.2 | 3.5d | 85.5 | 8 | 88.0 | 0.8Le | 84.7 | 0.6D | 84.4 | 0.8D | 86.1 |
| 5 | 18° | 82.8 | 0.9D | 85.3 | 3.0D | 81.9 | 0.8D | 81.4 | 3.0D | 83.8 | 3cm | 85.1 | 4.0d | 84.9 | 9 | 84.0 | 0.9Le | 88.7 | 0.8D | 82.8 | top | 84.3 |
| optimal parameter value | 12° | | 0.8D | | 0.5D | | 0.4D | | 2.0D | | 2cm | | 3.0d | | 8 | | 0.7Le | | 0.5D | | 0.8D | |
