# Supplementary material for: Numerical Simulation and Structural Optimization of the Inclined Oil/Water Separator
Source: PLoS One. 2015 Apr 13;10(4):e0124095. doi: 10.1371/journal.pone.0124095 (PMC4395151; doi:10.1371/journal.pone.0124095)
Supplement: S6 Table — (DOC) [file pone.0124095.s006.doc]

**Table S6: The effect of inclination on oil/water separation efficiency**

| Inclination | Sample | Water content in the liquid | Water content at the oil outlet | Oil content at the water outlet | Average water content | Average water content at the oil outlet | Average oil content at the water outlet | Separation efficiency | | |
| --- | --- | --- | --- | --- | --- | --- | --- | --- | --- | --- |
|  |  |  |  |  |  |  |  | 15#Station | 20# Station | Simulation value |
| 0° | 1 | 90.2% | 57.5% | 0.85% | 90.7% | 58.2% | 0.837% | 81.25% | 91.04% | 85.80% |
|  | 2 | 90.5% | 58.3% | 0.82% |  |  |  |  |  |  |
|  | 3 | 91.3% | 58.8% | 0.84% |  |  |  |  |  |  |
| 9° | 1 | 91.0% | 43.2% | 0.38% | 90.8% | 43.7% | 0.363% | 89.52% | 96.06% | 92.32% |
|  | 2 | 90.8% | 44.1% | 0.36% |  |  |  |  |  |  |
|  | 3 | 90.5% | 43.8% | 0.35% |  |  |  |  |  |  |
| 12° | 1 | 90.5% | 29.8% | 0.28% | 90.6% | 28.8% | 0.297% | 92.35% | 96.84% | 95.31% |
|  | 2 | 90.8% | 28.5% | 0.30% |  |  |  |  |  |  |
|  | 3 | 90.5% | 28.2% | 0.31% |  |  |  |  |  |  |
| 15° | 1 | 90.6% | 32.8% | 0.32% | 90.8% | 32.2% | 0.337% | 90.21% | 96.34% | 93.48% |
|  | 2 | 90.8% | 31.6% | 0.34% |  |  |  |  |  |  |
|  | 3 | 91.0% | 32.2% | 0.35% |  |  |  |  |  |  |
