# Supplementary material for: Numerical Simulation and Structural Optimization of the Inclined Oil/Water Separator
Source: PLoS One. 2015 Apr 13;10(4):e0124095. doi: 10.1371/journal.pone.0124095 (PMC4395151; doi:10.1371/journal.pone.0124095)
Supplement: S7 Table — (DOC) [file pone.0124095.s007.doc]

**Table S7: The effect of residence time on oil/water separation efficiency**

| Residence time | Sample | Water content in the liquid | Water content at the oil outlet | Oil content at the water outlet | Average water content | Average water content at the oil outlet | Average oil content at the water outlet | Separation efficiency | |
| --- | --- | --- | --- | --- | --- | --- | --- | --- | --- |
|  |  |  |  |  |  |  |  | experimental value | Simulation value |
| 5min | 1 | 90.4% | 40.2% | 0.42% | 90.43% | 39.97% | 0.423% | 95.57% | 91.30% |
|  | 2 | 90.6% | 40.5% | 0.45% |  |  |  |  |  |
|  | 3 | 90.3% | 39.2% | 0.40% |  |  |  |  |  |
| 6min | 1 | 90.5% | 27.8% | 0.31% | 90.63% | 27.70% | 0.307% | 96.73% | 95.31% |
|  | 2 | 90.8% | 28.2% | 0.32% |  |  |  |  |  |
|  | 3 | 90.6% | 27.1% | 0.29% |  |  |  |  |  |
| 7min | 1 | 91.0% | 23.5% | 0.23% | 90.63% | 23.70% | 0.23% | 97.54% | 95.45% |
|  | 2 | 90.5% | 24.0% | 0.22% |  |  |  |  |  |
|  | 3 | 90.4% | 23.6% | 0.24% |  |  |  |  |  |
| 8min | 1 | 90.7% | 19.7% | 0.23% | 90.63% | 19.23% | 0.220% | 97.65% | 95.73% |
|  | 2 | 90.4% | 19.2% | 0.23% |  |  |  |  |  |
|  | 3 | 90.8% | 18.8% | 0.20% |  |  |  |  |  |
| 9min | 1 | 90.5% | 18.2% | 0.19% | 90.50% | 18.13% | 0.207% | 97.82% | 95.96% |
|  | 2 | 90.3% | 18.1% | 0.23% |  |  |  |  |  |
|  | 3 | 90.7% | 18.1% | 0.20% |  |  |  |  |  |
| 10min | 1 | 90.6% | 18.0% | 0.22% | 90.57% | 18.07% | 0.203% | 97.84% | 96.12% |
|  | 2 | 90.8% | 18.6% | 0.21% |  |  |  |  |  |
|  | 3 | 90.3% | 17.6% | 0.18% |  |  |  |  |  |
